# In Graves’ disease, thyroid autoantibodies and ultrasound features correlate with distinctive histological features

**DOI:** 10.1007/s40618-023-02044-0

**Published:** 2023-02-25

**Authors:** A. Brancatella, L. Torregrossa, N. Viola, D. Sgrò, M. Casula, F. Basolo, G. Materazzi, M. Marinò, C. Marcocci, F. Santini, F. Latrofa

**Affiliations:** 1 Endocrinology Unit, Department of Clinical and Experimental Medicine, University Hospital of Pisa, Pisa, Italy; 2 Pathology Unit, Department of Surgical, Medical, Molecular Pathology and Critical Area, University of Pisa, 56126 Pisa, Italy; 3 Surgery Unit, Department of Surgical, Medical, Molecular Pathology and Critical Area, University of Pisa, 56126 Pisa, Italy

**Keywords:** Hyperthyroidism, Pathology, TSH receptor antibodies, Thyroglobulin antibodies, Thyroperoxidase antibodies, Graves’ disease

## Abstract

**Purpose:**

Laboratory, imaging, and pathological features of Graves’ disease (GD), although well characterized, have been barely correlated each other. Aim of the study was to link laboratory and ultrasound characteristics of GD with its pathological features.

**Methods:**

We correlated laboratory and ultrasound data at the time of diagnosis in 28 consecutive GD patients who underwent thyroidectomy with their pathological features, i.e., lymphocytic infiltration and follicular hyperplasia (both classified as mild or severe).

**Results:**

Thyroid volume correlated positively with the levels of FT4 (*P* = 0.002, *r*^2^ = 0.42), FT3 (*P* = 0.011, *r*^2^ = 0.22), autoantibodies to thyroglobulin (TgAbs) (*P* = 0.016, *r*^2^ = 0.32), autoantibodies to thyroid peroxidase (TPOAbs) (*P* = 0.011, *r*^2^ = 0.34) and the extent of lymphocytic infiltration (*P* = 0.006 comparing mild to severe lymphocytic infiltration) but not with the levels of autoantibodies to the thyrotropin receptor (TRAbs) and to follicular hyperplasia. Compared to subjects with mild lymphocytic infiltration, those with severe lymphocytic infiltration showed higher levels of TgAbs (316 vs 0.0 IU/mL, *P* < 0.0001) and TPOAbs (295 IU/mL vs 14 IU/mL, *P* < 0.0001) and similar levels of TRAbs (7.5 vs 13 IU/mL, *P* = 0.68). Compared to patients with mild, those with severe follicular hyperplasia had similar levels of TgAbs (76 vs 30 IU/mL, *P* = 0.31) and TPOAbs (251 IU/mL vs 45 IU/mL, *P* = 0.26) but higher levels of TRAbs (39 vs 7.2 IU/mL, *P* < 0.001).

**Conclusion:**

In GD, TgAbs and TPOAbs levels correlate with the extent of lymphocytic infiltration, TRAbs levels with the degree of follicular hyperplasia. Thyroid volume, the main factor influencing the severity of hyperthyroidism, is related to lymphocytic infiltration and not to follicular hyperplasia.

## Introduction

Graves’ disease (GD) is an autoimmune disorder which recognizes autoantibodies to the thyrotropin receptor (TRAbs) as the pathogenic driver [1–3]. At variance with many autoimmune diseases in which autoantibodies cause tissue damage, TRAbs stimulate the thyroid, inducing goiter and hyperthyroidism [2]. Patients with GD experience stigmata of hyperthyroidism, namely tachycardia, anxiety, insomnia and weight loss [1]. Currently, Graves’ orbitopathy occurred in up to 30% of patients whereas pretibial myxedema is reported in less than 10% of GD patients [4]. A diffuse goiter with high vascularization at neck ultrasound and high uptake at thyroid scintigraphy are the typical features of GD [1]. At histology, thyroid from GD patients is characterized by lymphocytic infiltration and follicular hyperplasia [5, 6]. In addition to TRAbs, the hallmark of the disease, autoantibodies to thyroglobulin (TgAbs) and to thyroid peroxidase (TPOAbs), which suggest a concomitant thyroiditis, are frequently detected in the serum of GD patients [7, 8]. The question whether GD always arises on a pre-existing thyroiditis or can develop because of the appearance of TRAbs on an otherwise normal thyroid remains unsettled.

Aim of the present study was to better characterize the features indicative of thyroid stimulation and those of lymphocytic thyroiditis in GD. For this purpose, we correlated the laboratory results and the ultrasound characteristics obtained at the time of diagnosis from a cohort of GD patients who underwent thyroidectomy with the histopathological features.

## Methods

### Study design and population

In 2017, seventy-four subjects underwent total thyroidectomy at the Endocrine Surgery Unit of University Hospital of Pisa because of GD. Among them, we identified the patients who had received a full evaluation, including blood tests and neck ultrasound, and the first diagnosis, prior to anti-thyroid treatment, at the Endocrinology Unit. Laboratory tests included measurement of FT4, FT3, TSH, TRAbs, TgAbs and TPOAbs. Twenty-eight patients matching the criteria were included in the study. The study was conducted in accordance with the ethical principles of Declaration of Helsinki. Data publication was approved by the local institutional review committee (Comitato Etico di Area Vasta Nord Ovest–CEAVNO). Patients were informed and gave their consent to participate in the study. All patients had been treated with anti-thyroid drugs prior to thyroidectomy.

### Laboratory testing

Thyroid hormones and TSH were tested by immunoenzymatic assays (Ortho-clinical diagnostic Inc., Rochester, NY). Reference ranges were 8.0–18.0 ng/dL for FT4, 2.5–5.0 ng/L for FT3 and 0.4–4.0 mIU/L for TSH, respectively. TgAbs and TPOAbs were measured by AIA-Pack 2000 (Tosoh Corporation, Tokyo, Japan); analytic, functional and positive cut-offs were 6 IU/mL, 8 IU/mL and 30 IU/mL for TgAbs and 6 IU/mL, 10 IU/mL and 20 IU/mL for TPOAbs. TRAbs were measured by ELISA (ElisaRSR™ TRAb 3^rd^ generation, Cardiff, UK) (positivity cutoff > 1.5 IU/mL).

### Thyroid imaging

Neck ultrasound was performed by Technos (Esaote Biomedica, Genova, Italy), with a 7.5-MHz linear transducer. Thyroid volume was calculated using the ellipsoid volume formula. The normal thyroid volume in the Italian reference population is 4.0–12.1 mL in women and 6.0–16.0 mL in men [9]. According to their echoic pattern, thyroids were classified as slightly hypoechoic or markedly hypoechoic.

### Pathology analysis

Hematoxylin and eosin-stained slides of thyroid specimens were collected and re-submitted to microscopic assessment by a pathologist expert in thyroid pathology who was masked to biochemical and imaging features of the patients and who carefully evaluated the histological features typical of Graves’ disease, i.e., follicular hyperplasia and lymphocytic infiltration. Sections 3–5 μm thick were deparaffinized in xylene, dehydrated, and processed using a diaminobenzidine detection system-BenchMark ULTRA IHC System (Ventana Medical Systems, Inc., Tucson, AZ). Lymphocytes were immunohistochemically identified by a monoclonal antibody—CD45 LCA-directed against an antigen located on their membrane. The number of lymphocytes was expressed as the sum of the cells in the most representative high power field (× 40 magnification) and lymphocytic thyroiditis was classified as mild, when the total number of lymphocytes was less than 50 per field or severe, when the total number was more than 50 per field [5, 10].

In absence of an universally accepted classification of follicular hyperplasia, the degree of follicular hyperplasia was categorized as mild, when thyroid follicles showed little variability in size and the pseudo-papillary projections were scattered and thin, or severe, when the thyroid tissue showed a diffuse crowding of the follicles, a variable degree of papillary structures, small-sized follicles and a scalloping of the colloid.

### Statistical analysis

Statistical data analysis was performed using SPSS 21 (IBM Corp., Armonk, NY). Data are reported as mean ± SD or median with interquartile range (IQR), as indicated. The Shapiro–Wilk test was used to assess normality of data distribution of continuous variables. Statistical tests used to compare groups included Student’s *t* test for normally distributed variables and Mann–Whitney *U* tests for variables with skewed distribution. The Chi-squared or the Fisher exact tests were used to compare counts and frequencies between groups for categorical variables, as appropriate. Pearson’s (R) and Spearman’s (ρ) correlation coefficients were used to quantify association for Gaussian and skewed continuous variables, respectively.

## Results

### Characteristics of the study population

General characteristics, laboratory exams, ultrasound and pathology features of the study population are summarized in Table 1. At diagnosis, all subjects were hyperthyroid with high FT4 and FT3 and undetectable TSH levels. The time from the diagnosis of Graves’ disease to total thyroidectomy was 12.1 (range 6–24 months). Before surgery, 23 of 28 patients were treated with methimazole and 4 with propylthiouracil. Fourteen subjects had one or more thyroid nodules. No difference in the overall thyroid volume comparing patients with and without thyroid nodule(s) was observed. Twelve thyroid nodules meet the ultrasound criteria for fine needle aspiration, resulting benign. At histology, 10 were hyperplastic nodules and 2 follicular adenomas. Before surgery, 25 patients were treated with Lugol’s solution for a median of 5 days (range 3–11 days).

**Table 1:** Characteristics of the study population (*n* = 28)

General characteristics	
Mean age—years (SD)	41 (± 11)
Female	17
Male	11
Median time from diagnosis to thyroidectomy—months (SD)	12.1 (8.0–23.2)
Surgical indication (%)	
Persistent hyperthyroidism	13
Graves’ orbitopathy	12
Planning for pregnancy	2
Methimazole intolerance	1
Laboratory exams at the onset of disease (normal range)	
FT4 (8–18 ng/dL)	22.3 (10.5–45.4)
FT3 (2.5–5 ng/L)	11.4 (6.9–14.3)
FT3/FT4 ratio	0.4 (0.36–0.42)
TSH (0.4–4 mIU/L)	0.01 (0.01–0.45)
TgAbs (< 30 IU/mL)	
Positive	13
Level	350 (185–481)
TPOAbs (< 10 IU/mL)	
Positive	22
Level	91.0 (40.7–298.2)
TRAbs < 1.5 IU/mL)	
Positive	28
Level	15.1 (5.7–30.6)
Neck ultrasound features at the onset of disease	
Thyroid volume (mL)	28.3 (19.4–48)
Echoic pattern	
Slightly hypoechoic	12
Frankly hypoechoic	16
Pathology features	
Lymphocytic infiltration	
Mild	16
Severe	12
Follicular hyperplasia	
Mild	21
Severe	7

FT4, FT3, TSH, FT3/FT4 ratio, TgAbs, TPOAbs, TRAbs, and thyroid volume are reported as median (25–75° percentile)

*FT4*  free thyroxine, *FT3* free triiodothyronine, *TSH* thyrotrophin, *TgAbs* thyroglobulin antibodies, *TPOAbs* thyroperoxidase antibodies, *TRAsb* TSH receptor antibodies

At pathology analysis, lymphocytic infiltration was mild in 16 and severe in 12 samples, follicular hyperplasia mild in 21 and severe in 7.

### Correlation between laboratory tests at the time of diagnosis

The levels of FT4 and FT3 at the time of diagnosis did not correlate with the levels of TgAbs, TPOAbs and TRAbs (*P* > 0.05 for all correlations, data not shown).

**Fig. 1:**
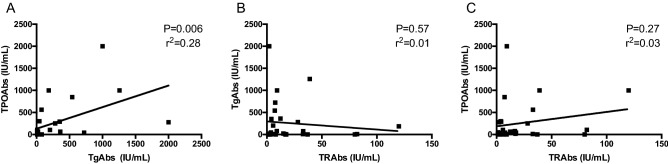
Correlations between TgAbs and TPOAbs (**A**), TRAbs and TgAbs (**B**), TRAbs and TPOAbs levels, (**C**) observed at the time of diagnosis in 28 GD patients

While TgAbs and TPOAbs levels correlated each other (*P* = 0.006, *r*^2^ = 0.28) (Fig. 1A), no correlations were observed between the levels of TRAbs and those of TgAbs and TPOAbs (*P* > 0.05) (Fig. 1B–C).

### Correlation between ultrasound and laboratory features at the time of diagnosis

**Fig. 2:**
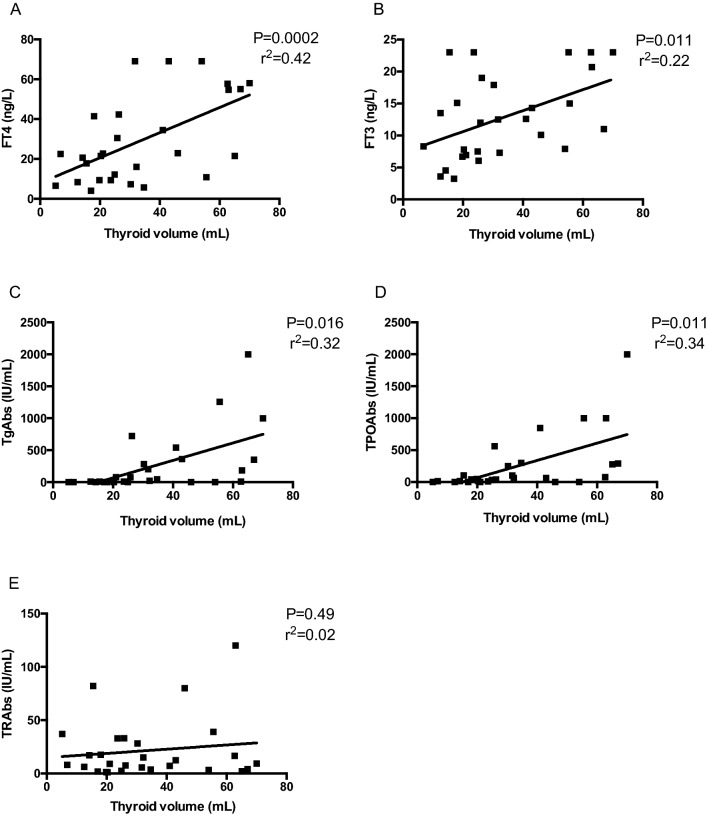
Correlations between thyroid volume at ultrasound and FT4 (**A**), FT3 (**B**), TgAbs (**C**), TPOAbs (**D**) and TRAbs levels (**D**) observed at the time of diagnosis in 28 GD patients

Thyroid volume positively correlated with the levels of FT4 (*P* < 0.005, *R*^2^ = 0.42), FT3 (*P* = 0.011, *r*^2^ = 0.22), TgAbs (*P* = 0.016, *r*^2^ = 0.32) and TPOAbs (*P* = 0.011, *r*^2^ = 0.34) (Fig. 2A–D) but not with the levels of TRAbs (*P* = 0.49) (Fig. 2E).

The levels of both TgAbs and TPOAbs were higher in subjects with a markedly hypoechoic than in those with a slightly hypoechoic gland (220 vs 20 IU/mL, *P* < 0.001 for TgAbs and 264 vs 12 IU/mL, *P* = 0.001 for TPOAbs). At variance, no difference in the levels of TRAbs were found comparing patients with a markedly hypoechoic to those with a slightly hypoechoic thyroid (8.3 vs 15.8 IU/mL, *P* = 0.58).

Patients with a markedly hypoechoic gland showed a higher thyroid volume compared to those with a slightly hypoechoic gland (33.2 vs 18.3 mL, *P* = 0.02).

### Correlations between the levels of thyroid hormones and thyroid antibodies, thyroid volume and histopathological features

FT4 and FT3 levels did not differ in patients with a mild lymphocytic infiltration compared to those with a severe lymphocytic infiltration (21.1 vs 32 ng/L, *P* = 0.5 for FT4 and 8.1 vs 13.2, *P* = 0.18 for FT3).

**Fig. 3:**
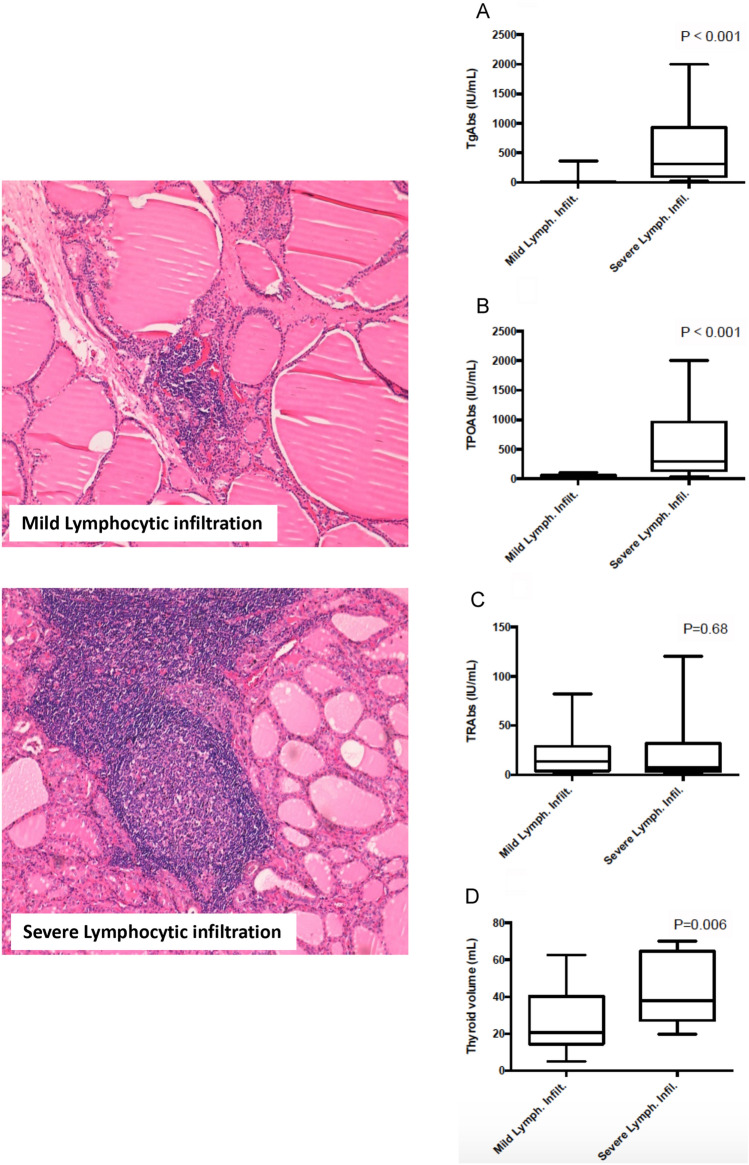
Histopathological images of mild and severe lymphocytic infiltration obtained from GD patients and correlation to laboratory and thyroid volume at ultrasound. An example of mild and one of severe lymphocytic infiltration of the thyroid are shown. In the specimen with a mild lymphocytic infiltration, lymphocytes are small in number and restricted to the interstitial stroma between the thyroid follicles. In that with a severe lymphocytic infiltration, the lymphocytic infiltrate is more prominent and is associated with lymphoid aggregate and germinal center (hematoxylin and eosin staining, original magnification × 100). TgAbs (**A**), TPOAbs (**B**), TRAbs levels (**C**) and thyroid volume (**D**) observed at the time of diagnosis in subjects with a mild and in those with a severe lymphocytic infiltration are compared.

Compared to subjects with a mild lymphocytic infiltration, patients with a severe lymphocytic infiltration had higher levels of TgAbs (316 vs 0.0 IU/mL, *P* < 0.0001) (Fig. 3A) and TPOAbs (295 IU/mL vs 14 IU/mL, *P* < 0.0001) (Fig. 3B), whereas no difference was observed in TRAbs levels (7.5 vs 13 IU/mL, *P* = 0.68) (Fig. 3C). Compared to subjects with a mild lymphocytic infiltration, patients with a severe lymphocytic infiltration had a higher thyroid volume (37.8 vs 25.6 mL, *P* = 0.06) (Fig. 3D). The median time from the diagnosis to thyroidectomy was similar in patients with mild lymphocytic infiltration compared to those with severe lymphocytic infiltration (10.8 vs 11.7 months, *P* = 0.39).

**Fig. 4:**
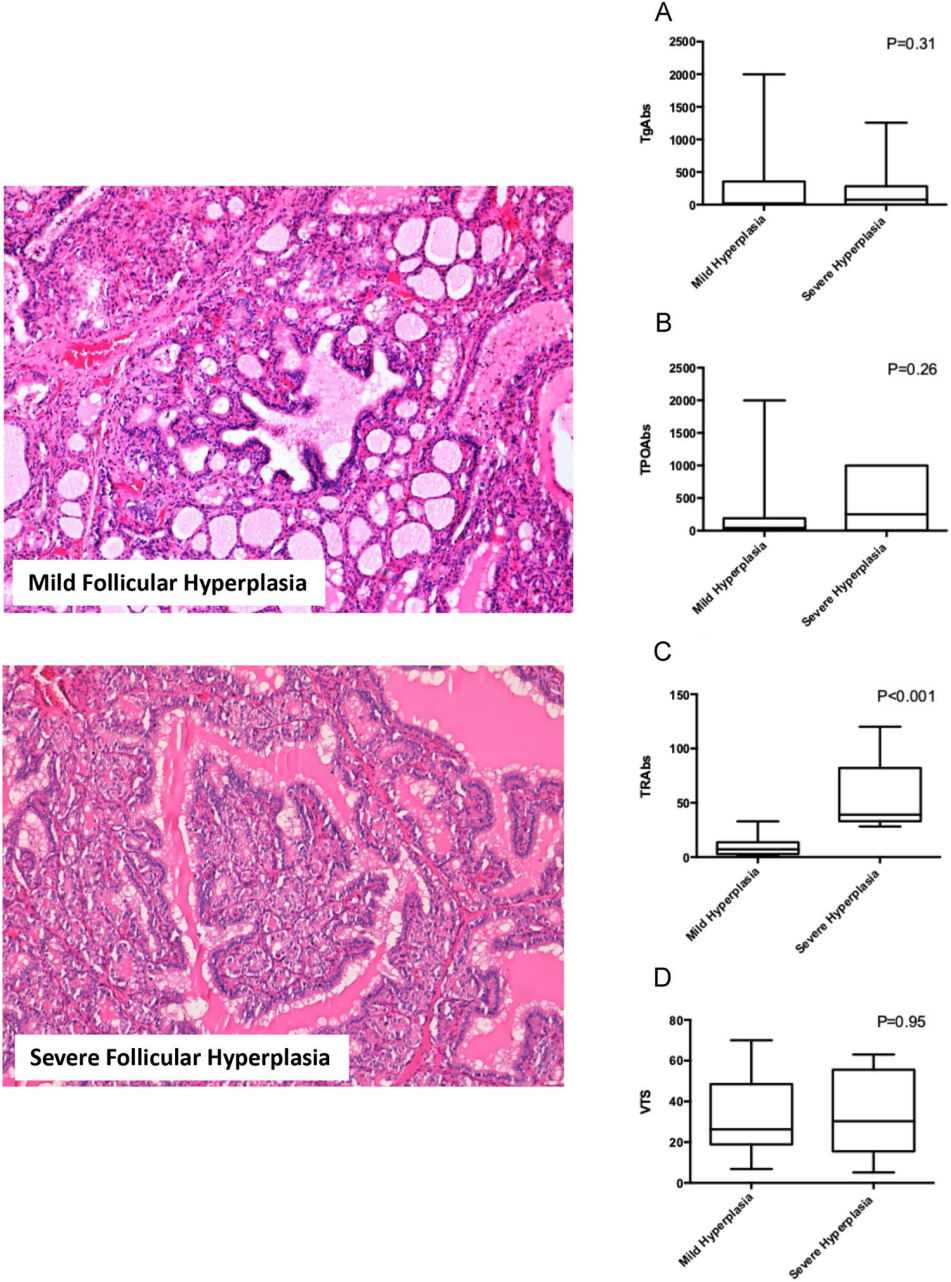
Histopathological images of mild and severe follicular hyperplasia obtained from GD patients and correlation to laboratory and thyroid volume at ultrasound. An example of mild and one of severe follicular hyperplasia of the thyroid are shown. In the specimen with mild follicular hyperplasia, thyroid follicles show little heterogeneity in size and pseudo-papillary projections are scattered and thin (hematoxylin and eosin staining, original magnification × 100). In that with severe follicular hyperplasia, papillary structures are exuberant and lined by cuboidal follicular cells showing round nuclei. TgAbs (**A**), TPOAbs (**B**), TRAbs levels (**C**) and thyroid volume (**D**) observed at the time of diagnosis in subjects with a mild and in those with a severe follicular hyperplasia are compared

FT4 and FT3 levels did not differ between subjects with a severe hyperplasia compared to those with a mild hyperplasia (17.8 vs 22.5 ng/L, *P* = 0.34 for FT4 and 12.1 vs 10.2 for FT3, *P* = 0.52). Compared to subjects with a mild follicular hyperplasia, patients with a severe follicular hyperplasia had similar levels of TgAbs (76 vs 30 IU/mL, *P* = 0.31) (Fig. 4A) and TPOAbs (25.1 IU/mL vs 45 IU/mL, *P* = 0.26) (Fig. 4B) and higher levels of TRAbs (39 vs 7.2 IU/mL, *P* < 0.001) (Fig. 4C). Compared to subjects with a mild follicular hyperplasia, patients with a severe follicular hyperplasia had a similar thyroid volume (30 vs 26 mL, *P* = 0.95) (Fig. 4D). The median time from the diagnosis to thyroidectomy was similar in patients with a mild follicular hyperplasia compared to those with a severe follicular hyperplasia (11.2 vs 13.1 months, *P* = 0.48).

## Discussion

Graves’ disease is the most common cause of hyperthyroidism in iodine sufficient areas, affecting 3% of women and 0.5% of men during their lifetime [1, 11]. Clinically, it is characterized at onset by symptoms and signs of thyrotoxicosis [1]. Patients may also experience extrathyroidal manifestations, namely Graves’ ophthalmopathy and dermopathy (pretibial myxedema) [12, 13]. At laboratory tests, GD patients show high levels of FT4 and FT3 and undetectable levels of TSH [1]. TRAbs are positive in most subjects [14, 15], whereas TgAbs are positive in up to 80% and TPOAbs in 90% of patients [7]. At imaging, GD is characterized by a diffuse goiter with high vascularization at neck ultrasound and high uptake at thyroid scintigraphy [1]. At pathology, follicular hyperplasia, the hallmark of the disease, is associated with a variable degree of lymphocytic infiltration [5, 6].

Few studies have investigated the link between laboratory results, ultrasound features and the pathological characteristics of GD [16, 17]. We decided to correlate the laboratory and imaging findings collected at the time of diagnosis from a cohort of GD patients, who underwent thyroidectomy, with their histopathological features.

The male to female ratio and the mean age of the study population were similar to those previously reported [1, 11]. All patients were hyperthyroid at the time of diagnosis, with a typical high FT3/FT4 ratio. TRAbs were positive in all subjects whereas TgAbs were positive in 46% and TPOAbs in 78%, respectively. A positive correlation was found between the levels of TgAbs and those of TPOAbs, whereas no correlation was found between the levels of TgAbs and TPOAbs and those of TRAbs.

TgAbs and TPOAbs but, to our surprise, not TRAbs levels positively correlated with thyroid volume and the degree of hypoechogenicity. At pathology, we observed that the severity of lymphocytic infiltration correlated with TgAbs and TPOAbs levels, while the severity of hyperplasia was positively associated with TRAbs levels.

All these findings are in agreement with the notion that two distinct, but related factors are involved in the pathogenesis of GD. On one hand, TRAbs are related to the follicular hyperplasia typical of the disease [2, 18] On the other hand, a “thyroiditis background” characterized by a diffuse lymphocytic infiltration and high levels of TgAbs and TPOAbs in observed in most GD thyroid glands [5]. TRAbs are required for the onset of GD but their levels do not correlate with the degree of lymphocytic infiltration [2, 19]. In animal models of GD thyroid lymphocytic infiltration is seldom observed after immunization with the TSHR [2, 19–21]. At variance, a strong association between lymphocytic infiltration and the levels of TgAbs and TPOAbs has been demonstrated in different settings of thyroid disease in humans [10, 22]. It has been shown that in GD lymphocytic infiltration is usually inhomogeneous and that the follicular hyperplasia is more extensive in the areas with a higher lymphocytic infiltration, likely because of a different degree of antigenicity between follicles [23, 24].

The pathogenic events underlying TRAbs appearance are still unknown. In our cohort, most patients had a thyroiditis background (i.e., lymphocytic infiltration and positive TgAbs and TPOAbs). However, lymphocytic infiltration was correlated to TgAbs and TPOAbs but not to TRAbs levels. The phenotype of positive TRAbs, negative TgAbs, negative TPOAbs and absent lymphocytic infiltration was observed in few patients. The appearance of TRAbs associated to the onset of GD after thyroid injury due to subacute thyroiditis or treatment with ^131^iodine is a rare event [25, 26]. Our data suggest that GD usually arises on a thyroiditis, linked to serum TgAbs and TPOAbs. The onset of GD because of the appearance of TRAbs in the absence of a lymphocytic thyroiditis is a rare occurrence.

In line with this view, we observed that, at the time of the onset of the disease, goiter was mainly due to lymphocytic infiltration and not to follicular hyperplasia, as commonly stated [3]. Indeed, to our great surprise, we did not observe the previously reported correlation between TRAbs levels and thyroid volume [27, 28]. This discrepancy is likely due to a variation in the phenotypic appearance of Graves’ disease, nowadays diagnosed earlier and in milder forms than in the past [23, 29]. It is indeed reasonable that the extensive follicular hyperplasia reported by the oldest studies and its correlation with TRAbs levels were due to a delay in diagnosis, which enabled a prolonged stimulation of TRAbs until thyroidectomy [28, 29]. At variance, in our patients who were thyroidectomized early after diagnosis, lymphocytic infiltration was the main determinant of thyroid volume. For the same reason we did not observe the previously reported correlation between the levels of TRAbs and those of FT3 [28, 30]. An alternative explanation for the last finding is that the TBI (thyrotrophin binding index) assay we used measure blocking in addition to stimulating TRAbs, which are those directly inducing hyperthyroidism. However, this is unlikely, because in GD total TRAbs measured as TBI are tightly associated to stimulating TRAbs [31, 32].

Because thyroidectomy is usually proposed to subjects with high thyroid volume, not eligible to radioiodine therapy, one could argue that the present study is affected by the bias of having included only patients with a large goiter [33]. However, it is noteworthy that the median thyroid volume of patients of the cohort is not particularly high. In addition, although we did not find any association between the timing from the diagnosis to thyroidectomy and the histological features, we do not exclude that the duration of the therapy with anti-thyroid drugs may change the follicular hyperplasia and/or the lymphocytic infiltration. This issue could be accurately evaluate only analyzing a large series of GD patients undergone total thyroidectomy at different times from the diagnosis.

In conclusion, our study highlights that GD usually arises from a pre-existing lymphocytic thyroiditis whereas the onset of TRAbs in its absence is uncommon. We observed that TRAbs levels are strictly related to the degree of follicular hyperplasia, TgAbs and TPOAbs levels to the amount of lymphocytic infiltration. Nowadays thyroid volume, the main factor influencing the severity of thyrotoxicosis at diagnosis, is mainly due to lymphocytic infiltration rather than to follicular hyperplasia.

## Data Availability

Some or all data generated or analyzed during this study are included in this published article or in the data repositories listed in References.
